# INTERDISCIPLINARY PERSPECTIVES ON CURRICULUM CONTENT FOR TRANSLATIONAL GEROSCIENCE EDUCATION

**DOI:** 10.1093/geroni/igae098.1338

**Published:** 2024-12-31

**Authors:** Iman Al-Naggar, George Kuchel, John Newman

**Affiliations:** Chair; Co-Chair; Discussant

## Abstract

Translational geroscience is a new field of study that aims to delay, prevent or alleviate several aging-related diseases simultaneously by targeting the so-called “biological hallmarks of aging,” a constellation of biological pathways at the root of natural aging and chronic conditions for which aging is the major risk factor (e.g., diabetes, cardiovascular disease, Alzheimer’s, cancer). Geroscience is a paradigm shift in healthcare because it aims to move away from single disease management to multi-disease prevention or alleviation and decrease the period of morbidity at the end of life. In order to train a workforce in conducting translational geroscience trials, the Geroscience Education and Training Network (NIH/NIA R25AG073119) is developing curricula towards a Certificate in Geroscience Research Program. This program is designed to train investigators in aspects specific to clinical trials of geroscience guided interventions (pharmaceutical or behavioral) in older adults. Evidence suggests that many factors often not considered in trial design play an important role in the outcomes of these trials; for example, social determinants of health contribute to accelerated aging and worse outcomes in minority populations, socially-disadvantaged persons, and other vulnerable populations, where chronic diseases arise at a much younger age. This symposium aims to begin a conversation between biological scientists, behavioral and social scientists and others to address the question “What knowledge from your specific field do translational geroscientists need to know to conduct meaningful and impactful geroscience clinical trials?” This insight is essential to help develop the curricula required to train a well-rounded workforce of translational geroscientists.

